# Numerical and Experimental Investigation on the Optical Manipulation from an Axicon Lensed Fiber

**DOI:** 10.3390/mi12020187

**Published:** 2021-02-12

**Authors:** Wu Zhang, Yanxiao Lin, Yusong Gao, Zekai Guo, Xiangling Li, Yuhong Hu, Pengcai Dong, Qifan Zhang, Xiaohui Fang, Meng Zhang

**Affiliations:** 1 School of Physics and Material Science, Guangzhou University, Guangzhou 510006, China; lyx180223@outlook.com (Y.L.); gys1919400074@outlook.com (Y.G.); gzk1919400022@outlook.com (Z.G.); 1919400035@e.gzhu.edu.cn (Y.H.); dpc1919400013@outlook.com (P.D.); zqf1919400060@outlook.com (Q.Z.); fangxiaohui@gzhu.edu.cn (X.F.); 2 School of Biomedical Engineering, Sun Yat-Sen University, Guangzhou 510006, China; lixling53@mail2.sysu.edu.cn; 3 Precision Medicine Institute, The First Affiliated Hospital of Sun Yat-Sen University, Sun Yat-Sen University, Guangzhou 510080, China

**Keywords:** optical force, axicon lensed fiber, tapered fiber, optical trapping

## Abstract

Here we numerically and experimentally studied the optical trapping on a microsphere from an axicon lensed fiber (ALF). The optical force from the fiber with different tapered lengths and by incident light at different wavelengths is calculated. Numerically, the microsphere can be trapped by the fiber with tapered outline $y=\pm \left|x\right|/0.5$ and $y=\pm \left|x\right|$ at a short incident wavelength of 900 nm. While for the fiber with tapered outline $y=\pm \left|x\right|/2$, the microsphere can be trapped by the light with longer wavelength of 1100 nm, 1300 nm, or 1500 nm. The optical trapping to a polystyrene microsphere is experimentally demonstrated in a microfluidic channel and the corresponding optical force is derived according to the fluid flow speed. This study can provide a guidance for future tapered fibre design for optical trapping to microspheres.

## 1. Introduction

Optical light carries momentum and therefore exerts forces on the objective in front of its propagation direction. This force is usually very weak that only until early 1900s scientists observed it experimentally [1]. Later due to the invention of laser, the measurement and regulation of optical force by scientific researchers began to develop rapidly. In 1970s, A. Ashkin and his colleagues carried out a study on the interaction force between photons and neutral atoms, and successfully restricted the motion of latex particles in water with light force, thus starting the application of light force in the microparticles [2,3]. In 1986, A. Ashkin focused a single beam of laser into an optical field with a high intensity gradient through a high numerical aperture objective lens, and realized the capture and movement of the micro-particle. This technique is then called optical tweezer [4]. Optical tweezer technology can capture objects with free contact and low pollution and damage, so it attracted wide research attention from the science community.

Optical tweezers technology has developed rapidly especially in the field of life science and microphysics. The invention of optical tweezers allows people to manipulate objects flexibly in the study of biological samples or micro-nano structures, rather than being restricted to passive observation. The optical tweezer force on micro-nano particles is usually free of contact and generally in the order of pico-or femto-Newton. Because it mainly relies on the light intensity rather than the light wavelength, it can realize a non-damage manipulation of living biological samples. Therefore, optical tweezers technology is widely applied to a variety of life science and bioengineering research fields. In the cell biology area, Ashkin et al. successfully demonstrated the trapping and dragging of cells by this technology at the beginning of the invention of optical tweezers [5]. Liang et al. combined optical tweezers and “light knife” to perform surgery on cells and organelles, including suspension, movement, sorting, and fusion, etc., and made quantitative measurement of the internal mechanical properties of cells and the interaction between cells [6]. Li and Tang et al. successfully reported the sorting [7] and sequencing [8] of a single chromosome. The rigidity caused by molecular shrinkage has also been accurately characterized by optical tweezers [9]. In single molecule biology, optical tweezers technology perfectly meets the research requirements of a single biological macromolecule due to its resolution of sub-nanometer precision and the pico-Newton resolution in the interaction of forces. Optical tweezers can even be used to measure the local molecular velocity in the dynamic molecular folding [10]. Optical tweezers are connected with biomolecules through microparticles, and molecules are analyzed in real time by observing the force between them, thus obtaining a variety of kinetic characteristics of single molecules [11,12]. Moreover, some scholars proposed to use optical tweezers to effectively control neural pathways [13]. In addition, researchers also captured and manipulated the microparticles in the colloids of soft substances by optical tweezers, and analyzed the internal properties of the colloids, such as viscosity [14,15]. In recent years, researchers also proposed the optical trapping [16,17] or sorting [18] through optical waveguides, which offers a new approach for single cell or bacterial study. With the in-depth development of research, the combined application of optical tweezers and other technologies has extended to more new research fields, which effectively promotes the development in the fields of life science, material science, physics, chemistry, medicine, and nanotechnology [19,20,21].

Some scientists also realized that it is possible to capture and manipulate microparticles by focusing the light with a tapered optical fiber [22,23,24,25]. Compared with the traditional optical tweezers technology, optical fiber tweezers have the advantages of smaller size, more flexibility, and convenience to operate. As early as 2006, Yuan et al. proposed a novel single tapered fiber optical tweezer by heating and drawing technology [23]. The microscopic particle trapping is numerically investigated and experimentally demonstrated. Liu et al. later proposed a double-tapered optical fiber tweezer through a chemical etching method, which traps yeast cells with high efficiency and non-contact capture [26]. In 2016, The B.J. Li team used single-fiber optical tweezers combined with high refractive index lens to capture particles of diameter below 100 nanometers [27]. A non-contact double-tapered optical fiber tweezers were also demonstrated later [26]. Today, optical tweezers are getting more and more attention. In this paper, we numerically studied the optical manipulation on a microsphere from an axicon lensed fiber (ALF) at different geometries and wavelengths. A fundamental analysis was carried out for the optical force by investigating the light intensity distribution. An experimental demonstration was also presented for the microsphere trapping in a microfluidic channel, and the corresponding optical force is derived. This study can provide a guidance for future lensed fiber design for optical trapping on microspheres.

## 2. Principle for Optical Force Calculation

The unit volume optical force on the microsphere can be derived from the electrical field and magnetic field distribution as

(1) f=ρE+J×B

where ρ and J are the net charge and current in the volume, ***E*** and ***B*** are the corresponding electrical and magnetic field. From Maxwell equation,

(2) $$\rho =\varepsilon_{0}\nabla \cdot E$$

and

(3) $$J=\;\frac{1}{\mu_{0}}\nabla \times B-\varepsilon_{0}\frac{\partial E}{\partial t}$$

***f*** can be derived as

(4) $$f=\varepsilon_{0}\left(\nabla \cdot E\right)E+\frac{1}{\mu_{0}}\left(\nabla \times B\right)\times B-\varepsilon_{0}\frac{\partial E}{\partial t}\times B$$

where

(5) $$\frac{\partial E}{\partial t}\times B=\frac{\partial }{\partial t}\left(E\times B\right)-\frac{\partial B}{\partial t}\times E$$

We know from Maxwell equation that

(6) $$\frac{1}{\mu_{0}}\left(\nabla \cdot B\right)B=0$$

and

(7) $$\nabla \times E=-\frac{\partial B}{\partial t}$$

Therefore, ***f*** can be derived as

(8) $$f=\varepsilon_{0}[\left(\nabla \cdot E\right)E+\left(E\cdot \nabla \right)E-\frac{1}{2}\nabla \left(E^{2}\right)]+\frac{1}{\mu_{0}}[\left(\nabla \cdot B\right)\cdot B+\left(B\cdot \nabla \right)B-\frac{1}{2}\nabla \left(B^{2}\right)]-\varepsilon_{0}\frac{\partial }{\partial t}\left(E\times B\right)$$

By introducing the Maxwell stress tensor $\overset{↔}{T}$ with element of

(9) $$T_{ij}=\varepsilon_{0}\left(E_{i}E_{i}-\frac{1}{2}\delta_{ij}E^{2}\right)+\frac{1}{\mu_{0}}\left(H_{i}H_{i}-\frac{1}{2}\delta_{ij}H^{2}\right)$$

which represents the *i* component of the electromagnetic momentum across the plane *j*, $\delta_{ij}$ is the delta function, it can be derived that

(10) $$f=\nabla \cdot \;\overset{↔}{T}-\frac{\partial g}{\partial t}$$

where $g=\varepsilon_{0}E\times B\;$ is the momentum density. Therefore, the total optical force on the microsphere is

(11) $$F=\;\oint_{s}\overset{↔}{T}dS-\frac{d}{dt}\oint_{V}gdV$$

g vanishes for a stable condition, and therefore the averaged optical force in a period of the field resonance can be expressed as

(12) $$\langle F\rangle =\;\langle \oint_{s}\overset{↔}{T}dS\rangle$$

It will be used for the optical force derivation in the section below. 

## 3. Results and Discussions

### 3.1. Axicon Lensed Optical Fiber Design

**Figure 1:**
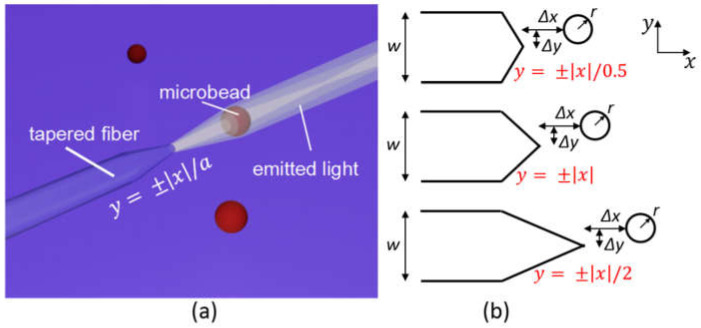
(**a**) The illustration of the axicon lensed fiber immersed in water and defined by a linear equation *y* = ±|*x*|/*a*; (**b**) the side view of the linearly tapered fiber with *a* = 0.5, 1, and 2, respectively.

Here we studied the optical manipulation on a microsphere based on an axicon lensed fiber (ALF) as shown in Figure 1. The outline of the ALF is characterized by a linear equation $y=\pm \left|x\right|/a$, where *a* is a constant value and defines the reciprocal of the taper slope. The fiber tapers from a diameter of *w* to a tip end. The tapered region has a length of *a* × *w*/2, which can be adjusted by changing the value of *a*. The diameter of the fiber is fixed at a constant value *w* = 20 µm and *a* is set at different values of 0.5, 1, and 2 for the optical manipulation comparison. The refractive index *n*_f_ of the ALF is set at 1.47. The fiber is immersed in water with refractive index of 1.33. It is assumed that only one microsphere appears in front of the ALF in the model. Here a polystyrene microsphere in diameter of 8 µm and refractive index of 1.58 is used for the study. The microsphere has a distance of Δ*x* to the fiber tip end in the *x* direction and Δ*y* to the fiber center axis in the *y* direction. The incident light will be focused by the tapered interface between the fiber and the water. Therefore, the microsphere in front of the ALF will suffer both gradient and scattering force.

### 3.2. Numerical Calculation and Analysis

**Figure 2:**
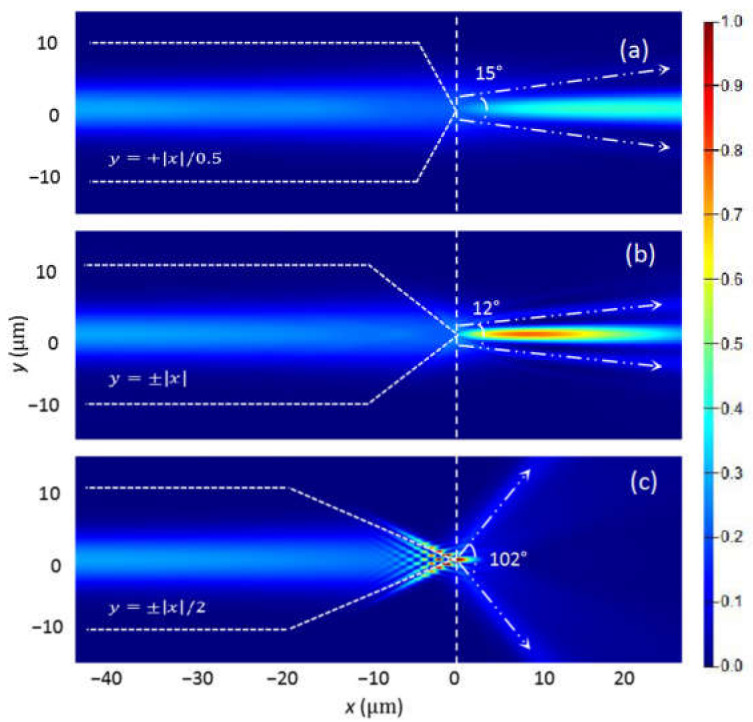
The light intensity distribution in the model for the axicon lensed fiber with the tapered outline equation of (**a**) *y* = ±|*x*|/0.5; (**b**) *y* = ±|*x*| and (**c**) *y* = ±|*x*|/2.

To analyze the optical force exerted on the microsphere by the light from the axicon lensed fiber, a two dimensional model is built and finite-difference time domain (FDTD) method is applied to calculate the electrical field and magnetic field in the model. A perfectly matched layer is set for the boundary condition of the calculation. A mesh grid of 0.1 µm is set in the whole calculation area and a finer mesh grid of 0.02 µm is then added in the 2 µm × 2 µm square area centered at the fiber tip end. The incident light is set as a Gaussian beam with beam waist of 4 µm and wavelength of 980 nm. The light intensity distribution from the ALF is calculated first as shown in Figure 2a–c by setting *a* = 0.5, 1, and 2 for the fiber tapered outline equation $y=\pm \left|x\right|/a$. Here the microsphere is not yet added in the model. The outline of each fiber in Figure 2 is illustrated by the short dash lines. A vertical dash line is used to better view the fiber tip, which is aligned with the origin points. When the ALF tapered equation is $y=\pm \left|x\right|/0.5$, the tapered length is 5 µm and the tapered angle is 127°. The light is focused near the paraxial axis and forms a typical Bessel beam with a diverging angle of 15°. When the ALF tapered equation is $y=\pm \left|x\right|$, the tapered length is 10 µm and the tapered angle is 90°. The focused Bessel beam is in a long area from *x* = 0 µm to 24 µm and the focal spot is centered at *x* = 8.3 µm. The diverging angle is estimated at 12°, indicating the light is more focused near the center axis. When the ALF tapered equation is $y=\pm \left|x\right|/2$, the tapered length is 20 µm and the tapered angle is 53°. A small focus spot is formed at *x* = 0 µm and interference pattern occurs in the tapered tip of fiber, which results a larger diverging angle of 102° and no Bessel beam is observed.

**Figure 3:**
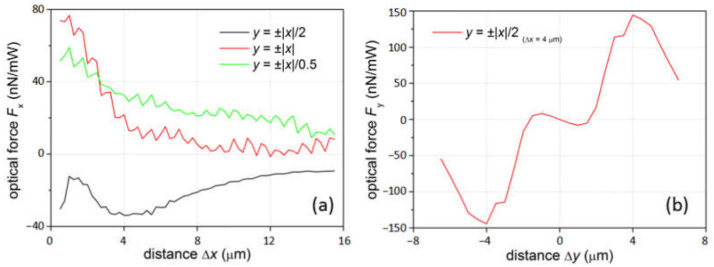
(**a**) The *x*-component of the optical force *F*_x_ on the microsphere at different distances to the fiber tip in the *x*-direction; (**b**) the *y*-component of the optical force *F*_y_ on the microsphere at different distances to the fiber tip in the *y*-direction when the fiber tapered outline is *y = ±|x|*/2.

**Figure 4:**
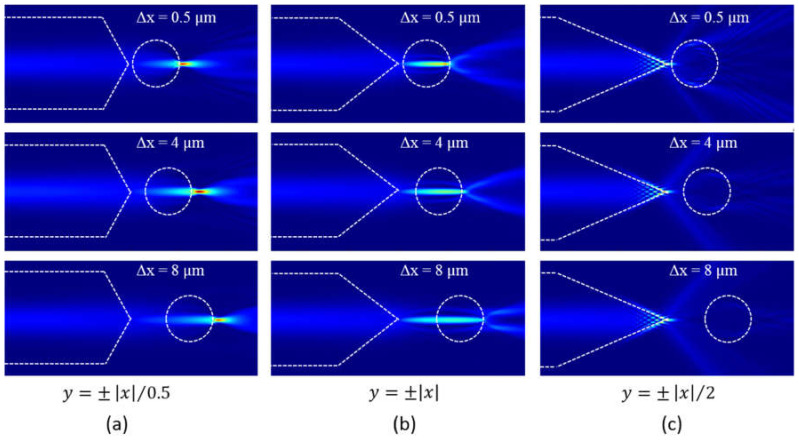
The light intensity distribution in the model consisting of the microsphere and the axicon lensed fiber with the tapered outline equation of (**a**) *y* = ±|*x*|/0.5; (**b**) *y* = ±|*x*|; and (**c**) *y* = ±|*x*|/2.

A microsphere was then added in the model at a distance of Δ*x* to the tip of the ALF in the *x*-direction, and Δ*y* to the fiber axis in the *y*-direction. We calculated the model with Δ*x* varying from 0.5 to 15.5 µm with an interval of 0.25 µm and Δ*y* fixing at 0. At each Δ*x*, the electrical field and the magnetic field of the model were calculated, and then substituted into Equation (9) for the Maxwell stress tensor calculation. An 8.4 µm × 8.4 µm square region concentric with the microsphere is defined as the closed surface for the Maxwell stress tensor integration using Equation (12). The *x*-component of the calculated optical force *F*_x_ on the microsphere is plotted in Figure 3a. For the fiber with tapered outline $y=\pm \left|x\right|/0.5$, *F*_x_ is always positive and the microsphere is pushed away from the fiber. Therefore, The *F*_x_ should be mainly due to the scattering force. *F*_x_ decreases slowly from 58 nN/mW to 21 nN/mW as Δ*x* increases from 0.5 µm to 8 µm, which can be analyzed by looking into the light intensity distribution as shown in Figure 4a for Δ*x* = 0.5 µm, 4 µm, and 8 µm, respectively. The light is highly focused in front of the microsphere, and the focal spot moves together with the microsphere as Δ*x* increases. Here the microsphere behaves as a lens and suffers the scattering force from the tapered fiber and gradient force by the focal spot. The two forces both point to the positive direction, and therefore push the microsphere away from the fiber.

For the fiber with tapered outline $y=\pm \left|x\right|$, *F*_x_ is also positive and decreases quickly from 76 nN/mW to almost 0 as Δ*x* increases from 0.5 µm to 8 µm. A new focal spot is formed to the right side of the microsphere center when Δ*x* = 0.5 µm as shown in Figure 4b. Therefore the microsphere suffers both positive scattering force from the tapered fiber and positive gradient force from the focal spot, and the total *F*_x_ is positive. When Δ*x* = 4 µm, the focal spot almost overlaps with the microsphere center, and the microsphere only suffers the scattering force from the fiber. When the microsphere moves further away to Δ*x* = 8 µm, the new focal spot is to the left side of the microsphere center. The gradient force due to the focal spot is then negative, which counteracts with the positive scattering force from the fiber. 

The resulted *F*_x_ is then closed to 0. For the fiber with tapered outline $y=\pm \left|x\right|/2$, *F*_x_ is always negative as shown in Figure 3a. The light intensity distribution as shown in Figure 4c is almost the same with that in Figure 2c and the focal spot is at the tip end of the fiber. As the beam diverges largely from the fiber to the water, the scattering force is small, while the gradient force pointing to the negative direction is relatively large due to the non-uniform light intensity distribution which is largest at the focal point. Therefore, a negative *F*_x_ is resulted and the microsphere is pulled close to the fiber. The *y*-component of the optical force *F*_y_ is then investigated when the microsphere moves vertically at different Δ*y* with a fixed Δ*x* = 4 µm for the case $y=\pm \left|x\right|/2$. As can be seen from Figure 2e, within the region between Δ*y* = -1 µm and 1 µm, *F*_y_ is a small negative value when the microsphere is at the positive side to the origin point, and vice versa. Therefore, the microsphere can be weakly confined within the region. This is due to the gradient of the focal spot. However, when |Δ*y*| goes beyond the region, the scattering force dominants and the microsphere will be pushed away from the fiber.

**Figure 5:**
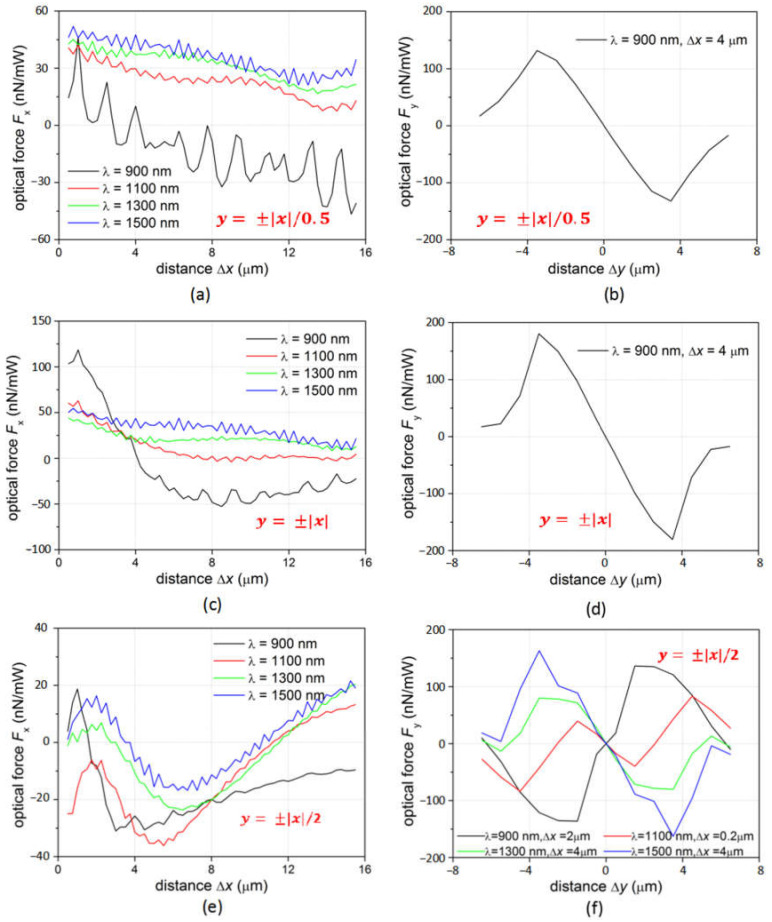
(**a**) The *x*-component of the optical force *F*_x_ and (**b**) the *y*-component of the optical force *F*_y_ by fiber *y* = ±|*x*|/0.5; (**c**) *F*_x_ and (**d**) *F*_y_ by fiber *y* = ±|*x*|; (**e**) *F*_x_ and (**f**) *F*_y_ by fiber *y = ±|x|*/2 on the microsphere at different incident wavelength.

The optical forces on the microsphere are also studied when Gaussian beam with different wavelengths is incident through the axicon lensed fiber. The *x*-component of optical force *F*_x_ at wavelength *λ* = 900, 1100, 1300, and 1500 nm is calculated with other parameters remaining the same. For the fiber with tapered outline $y=\pm \left|x\right|/0.5$, as plotted in Figure 5a, *F*_x_ is always positive for all Δ*x* when *λ* = 1100, 1300, and 1500 nm, which pushes the microsphere away from the fiber. While for the short wavelength *λ* = 900 nm, *F*_x_ is positive when Δ*x* < 4 µm and pushes the microsphere away from the fiber. The force then becomes negative when Δ*x* > 4 µm, indicating the microsphere can be trapped at Δ*x* = 4 µm position along the *x*-direction. The *y*-component of the optical force *F*_y_ is then investigated for the microsphere at different Δ*y* with Δ*x* = 4 µm and *λ* = 900 nm. As shown in Figure 5b, negative *F*_y_ is obtained for 3 µm > Δ*y* > 0, and vice versa. Therefore, the microsphere can be trapped stably at the position Δ*x* = 4 µm and Δ*y* = 0 µm. Similar forces are observed for the fiber with tapered outline $y=\pm \left|x\right|$ as shown in Figure 5c,d. The microsphere is also trapped by the 900 nm wavelength incident Gaussian beam at the position Δ*x* = 4 µm and Δ*y* = 0 µm. The light of the wavelength *λ* = 1100, 1300, and 1500 nm will push the microsphere away from the fiber due to the positive *F*_x_ at all Δ*x* position. For the fiber with tapered outline $y=\pm \left|x\right|/2$, as illustrated by the black line in Figure 5e, *F*_x_ is positive for Δ*x* < 2 µm and negative for Δ*x* > 2 µm when *λ* = 900 nm, therefore can trap the microsphere at Δ*x* = 2 µm in the *x*- direction. However, the calculated *F*_y_ at the Δ*x* = 2 µm, presented by the black line in Figure 5f, is negative for Δ*y <* 0 and positive for Δ*y* > 0. Therefore, the microsphere cannot be trapped in the *y*-direction at *λ* = 900 nm. At *λ* = 1100 nm, as indicated by the red line in Figure 5e, *F*_x_ is negative for Δ*x <* 10 µm, which will pull the microsphere all the way to the fiber. The *F*_y_ at the position with small Δ*x* = 0.2 µm and different Δ*y* is investigated as shown in the red line in Figure 5f. The region between Δ*y* = −1.5 µm and Δ*y* = 1.5 µm shows a negative slope, indicating the microsphere can be trapped close to the fiber tip. While for the model at *λ* = 1300 and 1500 nm, *F*_x_ is positive for Δ*x* < ~4 µm and negative for ~4 µm *<*Δ*x* < ~10 µm (by keeping Δ*y* = 0). The slope of *F*_y_ is negative for ~−3.5 µm < Δ*y* < ~3.5 µm. Therefore, the microsphere can be trapped at the position of Δ*x* = 4 µm and Δ*y* = 0.

### 3.3. Tapered Fiber Fabrication and Experimental Results

**Figure 6:**
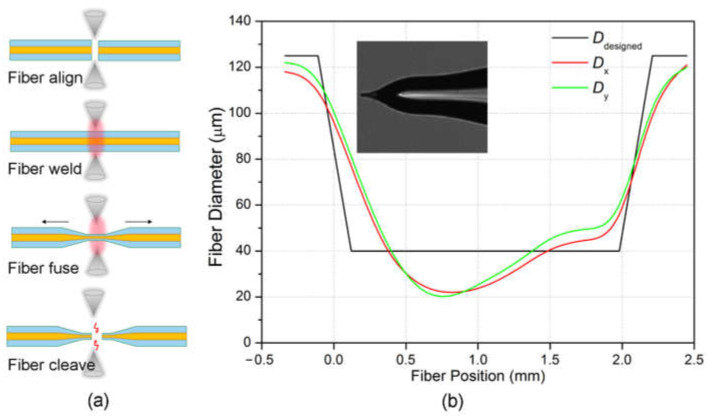
(**a**) Illustration of the tapered fiber fabrication process; (**b**) the pre-set tapered fiber diameter D_designed_ in the splicer and the pulled fiber diameters *D*_x_ and *D*_y_ before cleaving. Insertion: the tip end of the optical tapered fiber.

We fabricated the optical tapered fiber using a fiber fusion splicer (Fujikura, FSM-100P+) and the process is illustrated in Figure 6a. Two single mode optical fiber were placed in the splicer and automatically aligned. The fibers were welded together into one by the electrode discharge. The welded fiber was then pulled slowly at the two ends as the discharge process continues. During the process, the fiber started to fuse which tapered at the discharged center. Finally, the fiber was cleaved into two by the electrode discharge, which left a tapered shape at the fiber ending. Here a single mode fiber with initial diameter of 125 µm is pulled by the slicer and the tapered diameter *D*_designed_ is pre-set as shown in the black line of Figure 6b. The waist of the pulled fiber is set at 1.86 mm in length and 40 µm in diameter. The tapered length is set at 0.23 mm on each pulling side. The electrode discharge current was set at 279 mA and we can see a gradual thinning of the fiber in the pulling process. The resulted diameter of the fused fiber changes along with the fiber length, and is measured in two orthogonal directions perpendicular to the fiber axis and noted as *D*_x_ and *D*_y_, respectively. As shown in the red and green lines in Figure 6b, the resulted diameter had a deviation with the designed parameter, which highly depended on the discharge current and pulling speed during the fusion. Additionally, *D*_x_ and *D*_y_ in two orthogonal directions were almost the same, as the fiber was rotated continuously during the electrode discharging. The fiber is then fused to broken with a further electrode discharging, and a tapered fiber is formed as shown in the insertion of Figure 6b. 

**Figure 7:**
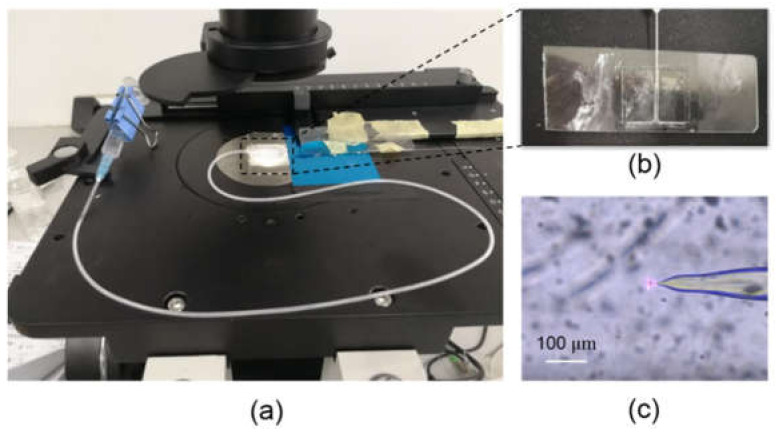
(**a**) Experimental setup for the tapered optical fiber trap in the microchannel chip; (**b**) the microchannel chip in which the tapered fiber is inserted; (**c**) the end tip of the tapered fiber with a polystyrene sphere being trapped at the tip.

To demonstrate the optical trap through the tapered optical fiber, we built an experimental setup as shown in Figure 7a. The fiber was integrated with a microchannel chip, in which the DI water fluid containing polystyrene spheres at a diluted concentration was injected through a tube-connected syringe. The microchannel chip was made by engraving a 0.5 mm wide channel on a 1-mm thick PMMA substrate, which is then bonded with a glass slide as shown in Figure 7b. The injection speed is simply controlled through the gravitation force by lifting the syringe at a fixed horizontal level. The diameter of the polystyrene sphere is 8 µm and their flow in the microchannel were recorded through a CCD camera on the microscope. A continuous laser at a wavelength of 980 nm and power of 300 mW is incident through the tapered fiber and a polystyrene sphere was trapped in front of the tapered fiber as shown in Figure 7c. The trapping effect is consistent with the calculated result when the fiber has a tapered outline of $y=\pm \left|x\right|/2$ at an incident light of 980 nm wavelength. The optical force pulled the microsphere all the way to the fiber and therefore confined it at the fiber tip. The flow speed v gradually increased as the syringe was slowly lift up. The sphere escaped out of the trap at the flow speed of 85 µm/s. The dragging force of the flow $F_{D}=-6\pi \eta rv$, where η is the viscosity of the DI water and equals to 1.005 × 10^−3^ kg/m·s, *r* is the radius of the sphere and equals to 4 µm. The dragging force $F_{D}$ was calculated as 6.44 pN, which equals to the optical force before the escape of the sphere from the optical trap. Compared with the results in Figure 3, the measured optical force is much smaller than the calculated force. The main reason to this difference is the fabricated fiber has a very small tapered slope and long tapered length which does not strictly follow the tapered outline of $y=\pm \left|x\right|/2$. Therefore the focus of the light is much weaker and the resulted pulling force on the microsphere will be much smaller. This can be further improved by optimizing the fiber fabrication process in future work. 

## 4. Conclusions

In conclusion, we numerically studied the optical manipulation on a microsphere from an axicon lensed fiber (ALF). The optical manipulation from fibers with different tapered slopes and different wavelengths is investigated. Numerically, the microsphere can be trapped by the fiber with tapered outline $y=\pm \left|x\right|/0.5$ and $y=\pm \left|x\right|$ at a short incident wavelength of 900 nm. While for the fiber with tapered outline $y=\pm \left|x\right|/2$, the microsphere is trapped by the light with longer wavelengths of 1100 nm, 1300 nm, and 1500 nm. An experimental demonstration is also presented for the microsphere trapping in a microfluidic channel. This study can provide a guidance for future lensed fibre design for optical trapping on microspheres.

## Data Availability

Not applicable.
